# Multiple Genome Sequences of the Important Beer-Spoiling Species *Lactobacillus backii*

**DOI:** 10.1128/genomeA.00826-16

**Published:** 2016-08-25

**Authors:** Andreas J. Geissler, Jürgen Behr, Rudi F. Vogel

**Affiliations:** a Technische Universität München, Lehrstuhl für Technische Mikrobiologie, Freising, Germany; b Technische Universität München, Bavarian Center for Biomolecular Mass Spectrometry, Freising, Germany

## Abstract

*Lactobacillus backii* is an important beer-spoiling species. Five strains isolated from four different breweries were sequenced using single-molecule real-time sequencing. Five complete genomes were generated, which will help to understand niche adaptation to beer and provide the basis for consecutive analyses.

## GENOME ANNOUNCEMENT

Beer is a selective environment for the growth of bacteria. Restrictive parameters in beer include ethanol, carbon dioxide, antibacterial hops, and anaerobicity. In addition, beer is characterized by a low pH (3.8 to 4.7) and a selective nutrient content (1–3). Nevertheless, lactic acid bacteria (LAB) of the genus *Lactobacillus* are capable of growing in and spoiling beer. Between 2010 and 2013, *Lactobacillus backii* caused 4.8 to 10% of all beer spoilage incidents in Germany, while spoiled beers are characterized by visible turbidity and slight acidification (4, 5). In order to gain insights into the genomic adaptation of *L. backii* to beer, we sequenced the complete genomes of five brewery isolates with the ability to spoil beer.

Beer spoilage ability was tested as described previously (6). High-molecular-weight DNA was purified from de Man, Rogosa, and Sharpe (MRS) liquid cultures using the Genomic-tip 100/G kit (Qiagen), as described previously (6). Single-molecule real-time sequencing (7) (PacBio RS II) was carried out at GATC Biotech (Konstanz, Germany). An insert size of 8 to 12 kb was selected for library creation, resulting in at least 200 Mb raw data from 1 to 2 SMRT cells (1 × 120-min movies) applying P4-C2 chemistry. Assembly was done with SMRT Analysis version 2.2.0.p2, using the Hierarchical Genome Assembly Process (HGAP) (8), and completed by manual curation (https://github.com/PacificBiosciences/Bioinformatics-Training/wiki/Finishing-Bacterial-Genomes). Genomes were annotated using the NCBI Prokaryotic Genome Annotation Pipeline (PGAP) and the Rapid Annotations using Subsystems Technology (RAST) server (9–11). Pan- and core genomes were calculated using CMG-Biotools and BADGE (6, 12).

Strain characteristics, sequencing statistics, genome information, and accession numbers are listed in Table 1. The chromosome sizes range from 2.55 Mbp to 2.67 Mbp, with G+C contents of 40.8 to 40.9%. We found seven to 10 plasmids (per strain) with G+C contents from 34.7 to 43.9%. Plasmid sizes range from 7,030 bp to 70,980 bp, resulting in overall genome sizes of 2.78 to 2.85 Mbp. The analysis of RAST-annotated genomes resulted in an *L. backii* core genome with 1,924 gene families and a pangenome with 2,889 gene families. The chromosomes encode five complete rRNA operons and 66 to 68 tRNAs.

**TABLE 1:** Strain characteristics, sequencing statistics, genome information, and accession numbers^a^

Strain	Source	BioSample no.	Accession no.	Avg coverage of HGAP assembly (×)	Size (Mbp)	No. of contigs	G+C content (%)	No. of PEGs	No. of CDSs
TMW 1.1988	Light wheat beer	SAMN04505726	CP014623 to CP014633	121	2.82	11	40.8	2,671	2,495
TMW 1.1989	Beer	SAMN04505727	CP014873 to CP014880	89	2.85	8	40.8	2,646	2,496
TMW 1.1991	Brewery environment	SAMN04505728	CP014881 to CP014889	99	2.82	9	40.7	2,590	2,437
TMW 1.1992	Brewery environment^b^	SAMN04505729	CP014890 to CP014898	109	2.78	9	40.8	2,621	2,450
TMW 1.2002	Brewery environment^b^	SAMN04505730	CP014899 to CP014906	168	2.84	8	40.7	2,653	2,478

a All strains (BioSamples) have beer spoilage ability and have been isolated from German breweries. All BioSamples are part of the BioProject PRJNA290141. Accession numbers are listed for all contigs of each whole genome (as range). Number of contigs are from chromosome plus plasmids and partial plasmids (only the case for TMW 1.1992). PEG, protein-encoding genes based on RAST annotation; CDS, coding sequences (coding) based on NCBI PGAP.

b TMW 1.1992 and TMW 1.2002 are from the same brewery.

The analysis of all five *L. backii* genomes revealed the presence of the same brewery-specific (99% sequence similarity, 99% coverage to each other) and plasmid-encoded fatty acid biosynthesis cluster as found in case of *Pediococcus damnosus* (6). Similarly, *L. backii* encodes an incomplete chromosomal fatty acid biosynthesis. Long-chain fatty acids are scarce in beer (13), while it was shown that the ability to produce fatty acids *de novo* is essential for *P. damnosus* growth in beer (6). The availability of these five *L. backii* genome sequences provides the basis for consecutive analyses (e.g., transcriptomics and plasmid curing experiments), with the objective to derive novel lifestyle genes of beer-spoiling *L. backii*. It will further help understand the role of plasmids for LAB niche adaptation.

### Accession number(s).

The five complete *L. backii* genomes have been deposited in DDBJ/EMBL/GenBank under the accession numbers stated in Table 1.
